# Estimating vertical ground reaction forces during gait from lower limb kinematics and vertical acceleration using wearable inertial sensors

**DOI:** 10.3389/fbioe.2023.1199459

**Published:** 2023-09-29

**Authors:** David Martínez-Pascual, José M. Catalán, Andrea Blanco-Ivorra, Mónica Sanchís, Francisca Arán-Ais, Nicolás García-Aracil

**Affiliations:** ^1^ Biomedical Neuroengineering Research Group, Robotics and Artificial Intelligence Unit, Bioengineering Institute, Miguel Hernandez University, Elche, Spain; ^2^ INESCOP Footwear Technology Center, Elda, Alicante, Spain

**Keywords:** biomechanical analysis, gait analysis, magneto-inertial devices, machine learning, vertical ground reaction force

## Abstract

One of the most important forces generated during gait is the vertical ground reaction force (vGRF). This force can be measured using force plates, but these can limit the scope of gait analysis. This paper presents a method to estimate the vGRF using inertial measurement units (IMU) and machine learning techniques. Four wearable IMUs were used to obtain flexion/extension angles of the hip, knee, and ankle joints, and an IMU placed over the C7 vertebra to measure vertical acceleration. We trained and compared the performance of two machine learning algorithms: feedforward neural networks (FNN) and random forest (RF). We investigated the importance of the inputs introduced into the models and analyzed in detail the contribution of lower limb kinematics and vertical acceleration to model performance. The results suggest that the inclusion of vertical acceleration increases the root mean square error in the FNN, while the RF appears to decrease it. We also analyzed the ability of the models to construct the force signal, with particular emphasis on the magnitude and timing of the vGRF peaks. Using the proposed method, we concluded that FNN and RF models can estimate the vGRF with high accuracy.

## 1 Introduction

The analysis of human kinematics and kinetics has great potential in the prevention of injuries (Padua and DiStefano, 2009), and the analysis or prediction of musculoskeletal disorders (Fineberg et al., 2013). The analysis of human kinematics requires the use of motion capture data. Computer vision techniques (Graci et al., 2012), but also wearable inertial sensors (Sy et al., 2020) can be used for estimating the angle of the human limbs. On the other hand, when performing a kinetic analysis, the human joint forces and torques are estimated by the forces exerted, using inverse dynamics methods (Moya-Angeler et al., 2017).

In a lower extremity biomechanical analysis, the kinetic data must include the ground reaction forces produced (Lencioni et al., 2019), which can be divided into the mediolateral ground reaction force, the anteroposterior ground reaction force, and the vertical ground reaction force (Kram et al., 1998). Vertical ground reaction force (vGRF) is the largest component of the ground-generated forces during gait. The vGRF is generated in the sagittal plane and represents the magnitude and pattern of mechanical loading in the vertical direction at the foot (Jacobs et al., 1972). During gait, three main peaks can be distinguished in this force (Marasović et al., 2009). First, the loading peak (LP) is produced, a local maximum corresponding to the loading response. Then, there is a local minimum corresponding to the mid-stance phase (MP). Finally, there is a second local maximum during the terminal stance phase (TP). The magnitude and timing of these peaks has influence in the lower limb joints and muscle loads. For this reason, the analysis of vGRF characteristic peaks could be essential to study musculoskeletal disorders (Shafizadegan et al., 2016).

The vGRF measurement is usually performed by employing force plates. The force plates can rest over the ground, but also they can be embedded in treadmills. These plates might limit the space for the gait analysis, and they also can be financially costly. As a consequence of these limitations, some authors have studied the possibility of estimating the vGRF.

To overcome these drawbacks, the use of inertial measurement units (IMU) to estimate vGRF has been investigated. E. Sahabpoor and A. Pavic showed that it is possible to estimate the vGRF with a single IMU, using the subjects’ body mass and vertical acceleration to estimate the vGRF exerted on the human body. (Shahabpoor and Pavic, 2018). The authors compared several locations and concluded that placing an IMU over the C7 vertebra was the better location to achieve the lowest error in the vGRF estimation. However, the method proposed by these authors is not able to determine the force produced on the right and left lower limbs.

The development and improvement of machine learning algorithms have led some authors to apply these techniques to analyze gait kinematics and estimate vGRF.

In (Choi et al., 2013; Oh et al., 2013) the authors used human body kinematics and artificial neural networks to perform vGRF prediction. However, the main disadvantage is that they used several infrared cameras to acquire motion capture data, so this method might also limit the analysis space. On the other hand, other authors preferred to place IMUs over the lower limbs to monitor human movement. X. Jiang et al. used a Random Forest model to estimate the vGRF during gait with a single IMU using acceleration and gyroscope data (Jiang et al., 2020). F. J. Wouda et al. also used IMU sensors over the lower limbs (pelvis and lower legs) (Wouda et al., 2018). These authors used an artificial neural network to estimate the knee angle and artificial neural networks to estimate the vGRF during running using the estimated knee angle and the vertical accelerations from the placed IMUs.

The scientific literature shows that progress in machine learning algorithms has made it possible to achieve good accuracy when estimating the vGRF by different approaches. When performing a biomechanical analysis, we usually want to study not only the kinetics but also the lower limb kinematics. Hence, we intend to develop a Machine Learning model that makes use of the lower limb joint kinematics to estimate the vGRF. Moreover, it has to be mentioned that we have employed a method to estimate the hip, knee, and ankle angles in the sagittal plane by employing IMUs, so the gait analysis could be performed in any situation.

Moreover, as mentioned before, apart from the lower limb kinematics, vertical acceleration has been used to estimate the vGRF since there is a closed relation between them. However, when including these features together, the contribution to the vGRF prediction of lower limb kinematics and vertical acceleration has not been analyzed.

This work aims to develop a system to perform biomechanical analysis of the lower limbs based on wearable inertial sensors. For this purpose, we have used a method to measure the angle of the lower limb joints, and we have trained and compared the performance of machine learning models to estimate the vGRF during gait. We have introduced the lower limb joint kinematics and vertical acceleration measured at the C7 vertebra as inputs of the machine learning models to estimate the vGRF. In addition, we have analyzed the contribution to the vGRF estimation of this set of features using different machine learning methods.

## 2 Materials and methods

### 2.1 Lower limb joints measurement

The method proposed by T. Seel et al. has been used to estimate the joint angles of the lower limbs during gait (Seel et al., 2014). The proposed method is based on the analysis of the movement of the joints and it allows obtaining the joint angles with high accuracy with IMUs even at low acquisition rates (from 40 Hz) (Seel et al., 2012). This section describes the method and details the calibration and angle measurement process.

#### 2.1.1 Identification of the joint axis and position coordinates

This method avoids developing an algorithm that assumes a position and orientation of the different IMUs concerning the user’s lower limb. Moreover, to estimate joint angles by this algorithm, we must use gyroscope and accelerometer data from the IMUs, so it does not depend on a uniform magnetic field. Hence, we assume we have two IMUs attached to the upper and lower segment for each joint, so we measure the accelerations $a_{1}\left(t\right),a_{2}\left(t\right)\in R^{3}$ and the angular rates $g_{1}\left(t\right),g_{2}\left(t\right)\in R^{3}$ at a sample period Δ*t*.

The gyroscope data are used to identify the direction vectors $j_{1},j_{2}\in R$ of the hip, knee, and ankle flexion/extension axis in the local coordinates of the IMUs. Geometrically, *g*_1_(*t*) and *g*_2_(*t*) differ only by the joint angular velocity and a rotation matrix, so their projections into the joint plane have the same lengths for each instant. This can be defined as:

$$\|g_{1}\left(t\right)\times j_{1}{\|}_{2}-\|g_{2}\left(t\right)\times j_{2}{\|}_{2}=0\;\forall t$$ (1)

where ‖ ⋅‖_2_ is the Euclidean norm.

Moreover, apart from the direction vectors, the joint center position in the local coordinates of the IMUs must be calculated. These vectors $o_{1},o_{2}\in R$ are constant and only depend on the mounting position and orientation over the leg segments. In order to calculate them, T. Seel et al. propose that the acceleration of each sensor can be thought as the sum of the center of the joint acceleration, and the acceleration due to the rotation of the sensor around the joint center. The acceleration of the joint center should be the same in both IMU local frames, which can be expressed as:

$$\|a_{1}\left(t\right)-\Gamma_{g_{1}\left(t\right)}\left(o_{1}\right){\|}_{2}-\|a_{2}\left(t\right)-\Gamma_{g_{2}\left(t\right)}\left(o_{2}\right){\|}_{2}=0\;\forall t$$ (2)

$$\Gamma_{g_{i}\left(t\right)}\left(o_{i}\right)≔g_{i}\left(t\right)\times \left(g_{i}\left(t\right)\times o_{i}\right)+{\dot{g}}_{i}\left(t\right)\times o_{i}\;,\;i=1,2$$ (3)

where $\Gamma_{g_{i}\left(t\right)}\left(o_{i}\right)$ is the radial and tangential acceleration due to the IMU rotation around the joint center.

It should be noted that the constraints defined must be fulfilled by any given motion of the joint. Hence, we can identify *j*_1_, *j*_2_, *o*_1_, and *o*_2_ by minimizing the left side of Eqs 1, 2.

To determine *j*_1_ and *j*_2_, if we restrict the axis to the unit length, the problem becomes four-dimensional and we can express *j*_1_ and *j*_2_ in spherical coordinates:

$$j_{1}=\left[\begin{array}{c} \cos\left(\phi_{1}\right)\cdot \cos\left(\theta_{1}\right)\\ \cos\left(\phi_{1}\right)\cdot \sin\left(\theta_{1}\right)\\ \sin\left(\phi_{1}\right) \end{array}\right],j_{2}=\left[\begin{array}{c} \cos\left(\phi_{2}\right)\cdot \cos\left(\theta_{2}\right)\\ \cos\left(\phi_{2}\right)\cdot \sin\left(\theta_{2}\right)\\ \sin\left(\phi_{2}\right) \end{array}\right]$$ (4)

where *ϕ*_*i*_ and *θ*_*i*_ are inclination and azimuth. We also can define the sum of squared errors for *N* samples as:

$$\Psi \left(\phi_{1},\theta_{1},\phi_{2},\theta_{2}\right)≔\overset{N}{\underset{i=1}{\sum }}{\left(\|g_{1}\left(t_{i}\right)\times j_{1}{\|}_{2}-\|g_{2}\left(t_{i}\right)\times j_{2}{\|}_{2}\right)}^{2}$$ (5)

and in the same manner, we can also obtain *o*_1_ and *o*_2_ by defining another sum of squared errors:

$$\Psi \left(o_{1},o_{2}\right)≔\overset{N}{\underset{i=1}{\sum }}{\left(\|a_{1}\left(t_{i}\right)-\Gamma_{g_{1}\left(t_{i}\right)}\left(o_{1}\right){\|}_{2}-\|a_{2}\left(t_{i}\right)-\Gamma_{g_{2}\left(t_{i}\right)}\left(o_{2}\right){\|}_{2}\right)}^{2}$$ (6)

To minimize Ψ(*ϕ*_1_, *θ*_1_, *ϕ*_2_, *θ*_2_) and Ψ(*o*_1_, *o*_2_) we have employed the Trust Region Reflective algorithm (Byrd et al., 1988), but the problem could be resolved by employing any other algorithm, e.g., a Gauss-Newton algorithm.

To obtain the hip, knee, and ankle joint position coordinates (*o*_1_, *o*_2_), before the experimental session started the users were told to perform circling arbitrary movements. Moreover, concerning the estimation of the joint axis coordinates (*j*_1_, *j*_2_), we got data from the users walking to identify the principal axis of motion, i.e., the flexion/extension movements of the hip, knee, and ankle.

#### 2.1.2 Flexion/extension angle measurement

Once we have successfully obtained the joint axis coordinates (*j*_1_, *j*_2_) and the joint position coordinates (*o*_1_, *o*_2_) for each user, we can estimate the angle of the joints with the placed IMUs. These angles are calculated from accelerations and angular rates. Using the gyroscope data, the flexion/extension angles can be estimated by integrating the difference of the angular rates around the joint axis:

$$\alpha_{\mathrm{gyr}}\left(t\right)=\int_{0}^{t}\left(g_{1}\left(\tau \right)\cdot j_{1}-g_{2}\left(\tau \right)\cdot j_{2}\right)d\tau$$ (7)

As T. Seel et al. mention, by knowing the axis coordinates we can apply methods that need the axes of the sensor coincide with the joint or the segment axes (Liu et al., 2009). First, we move the measured accelerations from the local IMU coordinates to the joint axis:

$${\tilde{a}}_{1}=a_{1}\left(t\right)-\Gamma_{g_{1}\left(t\right)}\left(o_{1}\right),\;\;{\tilde{a}}_{2}=a_{2}\left(t\right)-\Gamma_{g_{2}\left(t\right)}\left(o_{2}\right)$$ (8)

The flexion/extension can be estimated by the angle between the projections of $\tilde{a_{1}}$ and $\tilde{a_{2}}$ into the joint plane. Then, we define a pair of joint plane axes $x_{1},y_{1},x_{2},y_{2}\in R^{3}$ for the local frames:

$$x_{1}=j_{1}\times c,\;\;y_{1}=j_{1}\times x_{1},\;\;x_{2}=j_{2}\times c,\;\;y_{2}=j_{2}\times x_{2}$$ (9)

where $c\in R^{3}$ could be any vector that makes none of the products zero. Finally, we can estimate the joint angle by accelerometer readings:

$$\alpha_{\mathrm{acc}}\left(t\right)=∠\left(\left[\begin{array}{c} \tilde{a_{1}}\left(t\right)\cdot x_{1}\\ \tilde{a_{1}}\left(t\right)\cdot y_{1} \end{array}\right],\left[\begin{array}{c} \tilde{a_{2}}\left(t\right)\cdot x_{2}\\ \tilde{a_{2}}\left(t\right)\cdot y_{2} \end{array}\right]\right)$$ (10)

This *α*_*acc*_ is not affected by drift as *α*_*gyr*_ is, since we have not employed any integration to compute the angle, but it is affected by the accelerometer noise. Moreover, despite the drift, *α*_*gyr*_ is precise in short time scales. Hence, it could be appropriate to combine *α*_*acc*_ and *α*_*gyr*_. We have used a complementary filter (Young, 2009) defined as:

$$\alpha \left(t\right)=\lambda \cdot \alpha_{\mathrm{acc}}\left(t\right)+\left(1-\lambda \right)\cdot \left(\alpha \left(t-\Delta t\right)+\alpha_{\mathrm{gyr}}\left(t\right)-\alpha_{\mathrm{gyr}}\left(t-\Delta t\right)\right)$$ (11)

To obtain flexion/extension data from the participants, we have used a sample period Δ*t* = 0.016 (60 Hz) and *λ* = 0.02.

The method proposed by T. Seel et al. has shown a root mean squared error (RMSE) of between 3 and 5° for the hip, knee, and ankle flexion/extension movements (Kumar et al., 2018). Therefore, we can assume that we are accurate enough to perform an adequate gait analysis and vGRF.

### 2.2 Experimental setup

**FIGURE 1:**
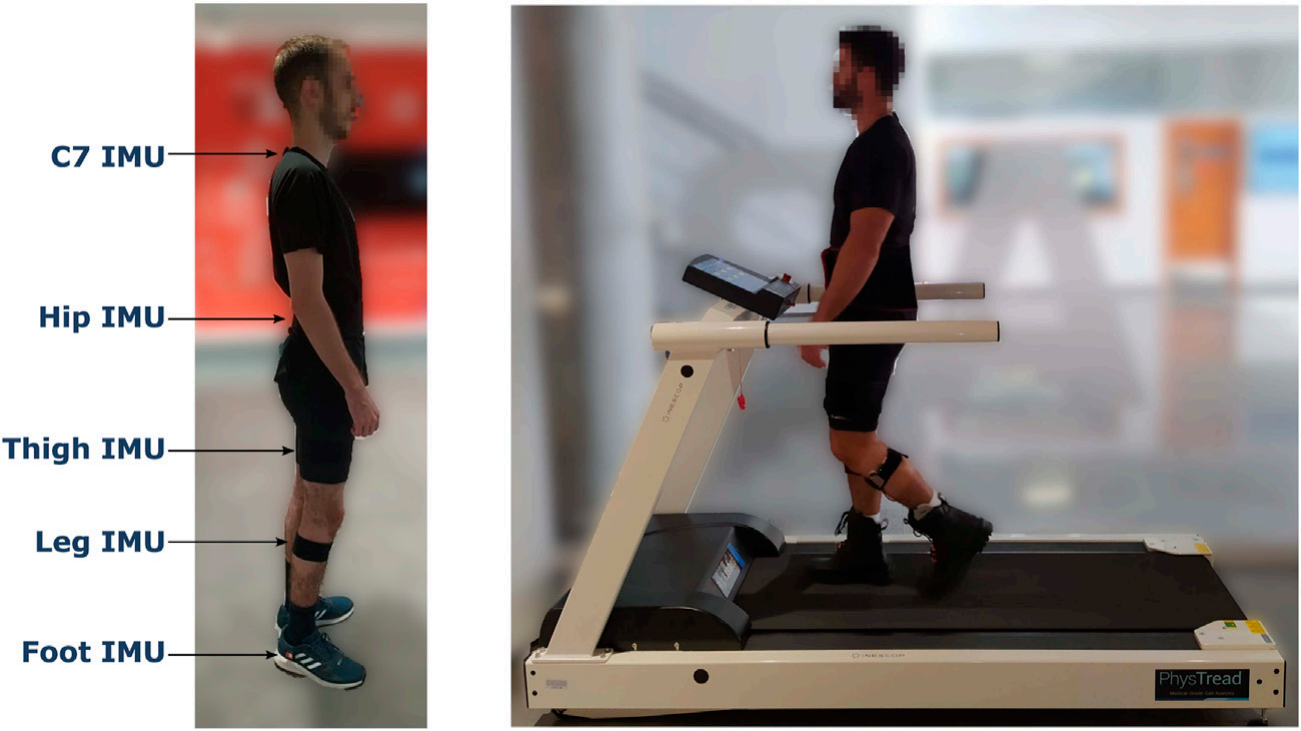
The devices employed during the experimental sessions. On the left image, the location of the IMUs placed to measure vertical acceleration (C7 IMU) and estimate the joint angles of the lower limb (hip, thigh, leg and foot IMUs). On the right image, the treadmill h/p/cosmos 150/50, used to acquire the vGRF during gait is shown.

The equipment used during the experimental sessions is shown in Figure 1.

Five XSens Dot IMUs were used to acquire kinematic data from the participants at a sampling rate of 60 Hz. Four inertial sensors were placed on the back of the hip, the thigh, the leg, and the foot to estimate the joint angles of the lower limb. The remaining IMU was placed over the C7 vertebra to provide vertical acceleration data. The hip, thigh, and leg IMUs were placed with elastic straps, and the C7 and foot sensors were attached to the skin and the user’s shoes with stickers.

A treadmill with an embedded force plate model h/p/cosmos 150/50 was used to measure the generated vGRF during gait. The vGRF data were recorded at 100 Hz.

### 2.3 Subjects

Twelve volunteer able-bodied subjects participants were involved in the experimental sessions, 10 male and 2 female. The ages were between 23 and 52 years old (29.8 ± 7.4), with heights ranging between 1.65 m and 1.87 m (176.2 ± 7.4 cm) and weights between 56.1 kg and 90.2 kg (76 ± 12.5 kg). Written informed consent was obtained from the individuals for the publication of any potentially identifiable images or data included in this article.

### 2.4 Experimental protocol

At the beginning of the experimental session, the IMUs were placed on the participants at the established locations, and the height and weight of users were measured. Once the user wore the sensors, we proceeded to obtain the calibration data to estimate the joint angles of the lower limb. First, the users were told to perform arbitrary circling movements with the hip, knee, and ankle to obtain the center position coordinates *o*_1_, *o*_2_ of each joint for 1 min. Then, the participant walked for 1 min over the treadmill at a comfortable speed to obtain the flexion/extension joint axis coordinates *j*_1_, *j*_2_.

After the calibration was carried out, the subjects walked over the treadmill at four different speeds selected to cover a wide range of normal gait speeds Weber (2016): 1.5 km/h, 2.5 km/h, 3.5 km/h, and 4.5 km/h. When the treadmill reached the desired speed, the kinematics and vGRF data were recorded for 5 min. Between each activity, 2 min were left for rest.

### 2.5 Acquired data and processing

**FIGURE 2:**
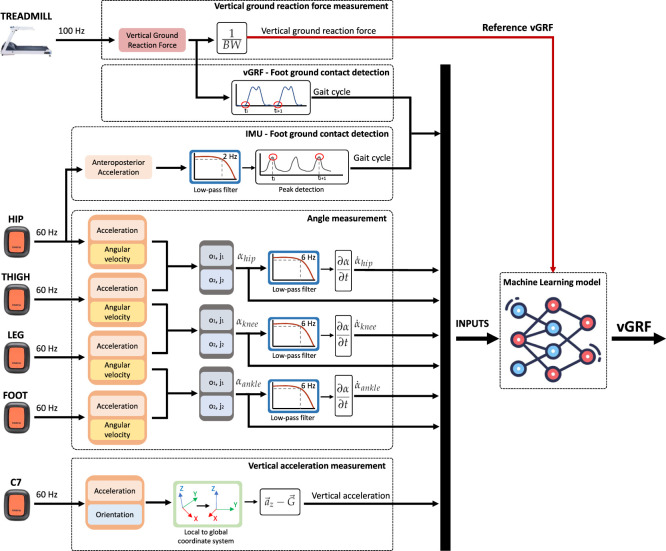
Signals obtained by the IMUs and the treadmill, and their processing before introducing them in the machine learning models. From the IMUs placed on the lower limbs, we have acquired acceleration and angular velocity to estimate the flexion/extension angles (T. Seel et al. method). With the IMU placed over the C7 vertebra, we measured the vertical acceleration. Moreover, the foot-ground contact has been detected using the anteroposterior acceleration measured at the hip to compute the gait cycle. The generated vGRF during gait has been measured and normalized by body weight. In the vGRF signal, the gait foot contact detection has been performed by finding the end of the stance phase (null force). The measured vGRF and the kinematics data have been synced by using the cycle gait. The flexion/extension joint angles and velocities, the vertical acceleration, and the cycle gait have been introduced as inputs of the models. The measured vGRF has been introduced as a reference for model learning.

The data acquired from each device and the signals processing to be used as the machine learning models inputs are detailed in this section and illustrated in Figure 2.

The vGRF was acquired from the plate-instrumented treadmill, which has been normalized by the body weight of the participants (BW).

From the hip, thigh, leg, and ankle IMUs, we acquired accelerometer and gyroscope data to estimate the lower limb joint angles in the sagittal plane. Once the hip, knee, and ankle angles were computed, we applied a forward-backward low-pass filter (Gustafsson, 1996) with a 6 Hz cutoff and we obtain the angular velocities of each joint.

To obtain the vertical acceleration we acquired the acceleration and orientation of the IMU placed over the C7 vertebra (Esser et al., 2009). First, by using the IMU orientation, we transform the acceleration vector from the local to the global coordinates system. Then, to obtain the vertical acceleration, we remove gravity from the measured vertical acceleration, which corresponds to the Z-axis acceleration in the global coordinates system.

In addition, it is usual to identify the onset and end of the gait cycle and transform the data from the temporal domain to the gait cycle domain (0%–100%) when performing a gait analysis. Hence, all signals have been transformed to the cycle gait domain. To detect the onset of the steps we have detected the foot-ground contact detection by two methods. To identify the foot contact in the IMU data (inputs of the model) we have made use of the hip acceleration (Zijlstra and Hof, 2003). We applied a forward-backward low-pass filter with a 2 Hz cutoff. In the filtered signal, we detect the local maxima, which correspond to the left and right ground foot contacts. As a matter of convention, we employed the right foot contact to calculate the gait cycle. Moreover, we detected the foot-ground contact when the force value starts growing in the vGRF signal.

Signal synchronization begins by detecting the first foot contact with the ground in both the vGRF and the IMU system. In addition, by detecting the ground-foot contact in the vGRF and the IMU system the onset and end of each step can be detected. For each step, each vGRF and lower limb kinematics data is assigned a label according to the gait instant in which it occurs (gait cycle percentage). Finally, to train the machine learning models, the vGRF and leg kinematics data are synchronized from the gait instant in which they occur, so that each lower limb kinematics data is assigned a vGRF value.

**FIGURE 3:**
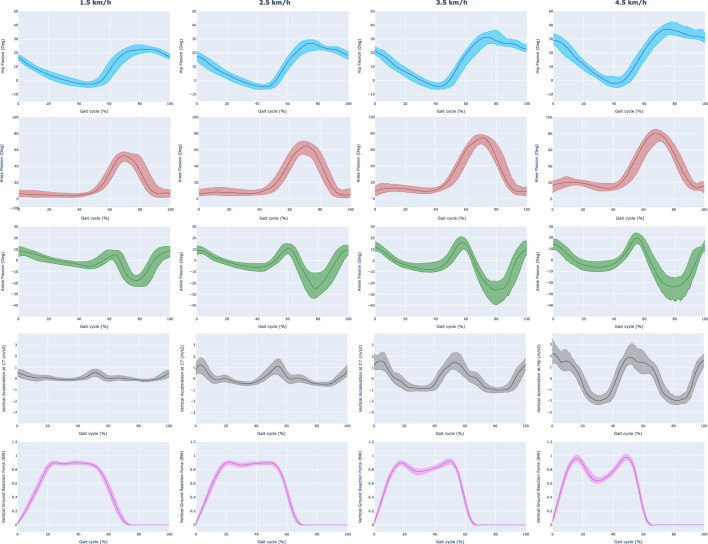
Acquired data during the experimental session. We have represented the hip, knee, and ankle flexion/extension angles (first, second, and third row), the vertical acceleration measured at the C7 vertebra (fourth row), and the measured vGRF (fifth row). The data is represented for different gait speeds (1.5–4.5 km/h) and normalized by the cycle gait.

The flexion/extension angles, the vertical acceleration, and the vGRF acquired are shown in Figure 3 for each gait speed. When the treadmill reached the desired speed, the kinematics and vGRF data were recorded for 5 min. Between each activity, 2 min were left for rest.

### 2.6 Machine learning models training

#### 2.6.1 Selected machine learning algorithms

Regression techniques can be used in order to estimate the vGRF. We decided to train Random Forest (RF) models and Feed-forward Neural Networks (FNN) since their use has been validated in previous studies by different authors.

The RF models consist of multiple decision trees (Zhang and Ma, 2012). These decision trees are trained independently with a data subset, and the output of the model will be determined by the best result given by the ensemble trees. This strategy allows the RF models to achieve accurate predictions as well as better generalizations, which come from the bagging scheme by decreasing variance.

The RF models have been trained with SciKit Learn Python Library (Pedregosa et al., 2011). RF training was performed with 170 decision trees, a tree maximum depth of 35, a minimum number of samples of 1, a minimum number of samples required to split an internal node of 2, and the number of inputs to consider when looking for the best split were calculated by the logarithm base 2 of the number of inputs.

The FNN models are a kind of artificial neural networks based on neurons that receive an input signal and process them by the activation function to an output signal (Svozil et al., 1997; Sharma et al., 2017). These neurons are ordered into layers, where the first layer is called the input layer, the last layer is called the output layer, and the layers between them are called the hidden layers. Each neuron of a particular layer is connected with all the neurons placed in the next layer, connected by the weights coefficients. The adjustment of the weights of the neural network is usually performed by back-propagation, employing a non-linear optimization method such as the gradient descent algorithm (Andrychowicz et al., 2016).

The FNN models have been trained with Keras Python Library (Gulli and Pal, 2017). We have employed FNN with one input layer of a number of neurons equal to the number of inputs, 5 hidden layers with 10 neurons, and an output layer with one neuron. We have used the Rectified Linear Unit (ReLu) as the activation function of the neurons, the Adam algorithm (Kingma and Ba, 2014) as the optimizer to adjust the network weights, and the RMSE as the loss function. To avoid overfitting, we have employed the dropout regularization method (Srivastava et al., 2014). Moreover, as recommended when using dropout, a constraint is imposed on the weights for each hidden layer. We have imposed that the maximum norm of the weights does not exceed a value of 4.

The hyperparameters tunned for both RF and FNN models are the best performing based on the RMSE with the training data.

#### 2.6.2 Preprocessing and training

For the training of the RF and FNN models, we introduced the joint angles, angular velocities, vertical acceleration, and cycle gait as inputs (Figure 2). In addition, the vGRF measured with the instrumented treadmill was introduced as the reference of the model.

We divided the data into 3 subsets to evaluate the performance of the models. First, we used data collected from 10 participants to train the model (intra-participants). This data collection was randomly divided into two subsets to validate the accuracy of the models by cross-validation: 80% of the data for training and the remaining 20% for validation. Data from 2 participants were also used to evaluate the performance of the models on users not involved in the learning process (inter-participants).

To train the models, 8 inputs were used: the flexion/extension angles and angular velocities of the hip, knee, and ankle; the vertical acceleration measured at the C7 vertebra; and the instant of the gait cycle.

It must be noted that we have scaled each input of the training data from its minimum and maximum, so we have numerical values between 0 and 1. We have also used the training scaler to evaluate the accuracy of the classifier with the test data.

As we introduced, one of the objectives of this work is to examine the contribution of vertical acceleration and lower limb kinematics in the vGRF estimation. To analyze the contributions, we realized two different pieces of training for each model.

• **Training 1 (Kinematics).** Joint angles, angular velocities, and cycle gait have been included as inputs of the models.
• **Training 2 (C7).** Joint angles, angular velocities, vertical acceleration, and cycle gait have been included as inputs of the models.

#### 2.6.3 Performance evaluation of the trained models

The performance achieved by the trained models has been analyzed as follows.

• **vGRF RMSE (BW).** A lower RMSE in the reproduction of the vGRF signal is understood as a better performance of the model.
• **Normalized RMSE (BW).** The Normalized RMSE (NRMSE) is defined as (Shahabpoor and Pavic, 2018):

$$NRMSE=\frac{RMSE}{\max vGRF_{\mathrm{measured}}-\min vGRF_{\mathrm{measured}}}$$ (12)

A lower normalized RMSE (NRMSE) in the construction of the vGRF is understood as a better performance of the model.

• **Correlation.** A higher correlation (*ρ*) between the measured and estimated vGRF signals is understood as a better performance of the model. To calculate the correlation, the Person’s correlation coefficient has been calculated.
• **LP, MP, and TP error (BW).** A lower error between measured and estimated vGRF peaks is understood as a better performance of the model. To find the LP and TP peaks we searched the local maximum in the first and second periods of the foot support. To calculate the MP peak we searched the local minimum between LP and TP peaks.
• **LP, MP, and TP delay (%).** A delay or advance close to 0 between measured and estimated vGRF peaks is understood as a better performance of the model. The delay is calculated as the difference between the estimated and measured peaks. A positive value means a delay of the estimated peak, and a negative value means an advance of the estimated peak to the measured peak.

#### 2.6.4 Statistical data analysis

We performed statistical data analysis to compare the accuracy of the models in estimating the vGRF with the validation and test data. First, we compared the four trained models (FNN-Kinematics, FNN-C7, RF-Kinematics, and RF-C7) in terms of RMSE. To perform a deep analysis of the vGRF signal construction, we have selected the best FNN and RF models, taking into account the evaluation detailed previously. For each model, we compared the vGRF estimation and peak reproduction with the validation and test data.

In the statistical analysis, first, a normality test was performed using the Kolmogorov-Smirnov test (Berger and Zhou, 2014). The results of the test showed that data distribution was not normal (*p* < 0.05).

The Friedman test was used to study the differences between the accuracy of the models (Sheldon et al., 1996). In the *post hoc* analysis, pairwise comparisons have been studied by the Wilcoxon signed-rank test with the zero method proposed by Pratt (1959).

## 3 Results

### 3.1 Feature importance

**FIGURE 4:**
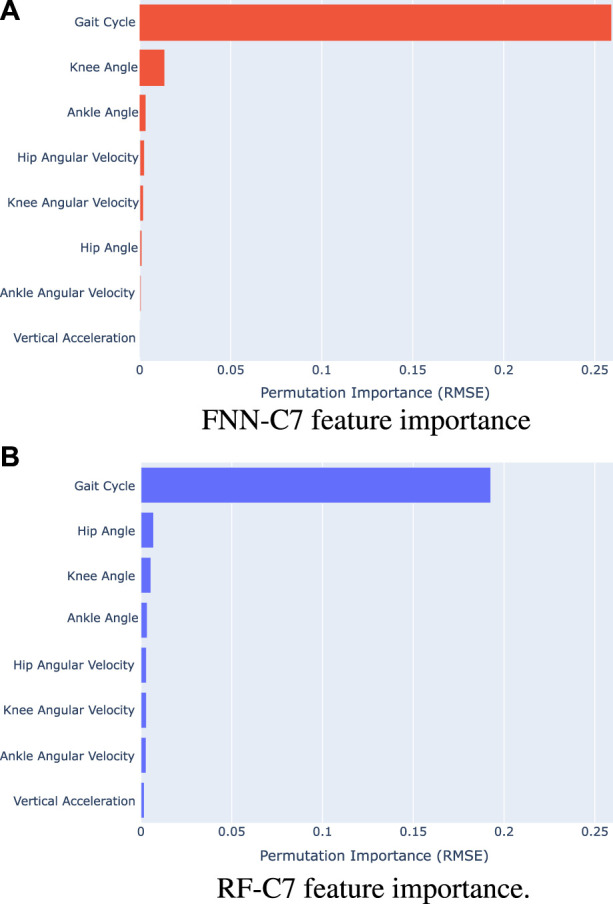
Feature importance computed with feature permutation **(A)** FNN-C7 model (Training 2) **(B)** RF-C7 model (Training 2)

To understand the influence of the angles, the angular velocities, the vertical acceleration, and the cycle gait in the training of the FNN and RF models, we have calculated the feature importance by feature permutation (Altmann et al., 2010). This method is defined to be the increase in the model error when a single feature is randomly shuffled, so we could understand how much the vGRF depends on each feature. The results obtained are shown in Figure 4, which shows the increase of the RMSE in the vGRF prediction when a feature is shuffled with the FNN-C7 model (Figure 4A and the RF-C7 model (Figure 4B).

The feature permutation results show that the gait cycle has the greatest influence on the performance of the models since the RMSE increases by 0.2590 BW in the FNN-C7 model and 0.1927 BW in the RF-C7 model. Concerning the lower limb kinematics, it can be observed that the knee and the ankle angles have the highest relevance on the FNN-C7, followed by the hip and knee angular velocity. By contrast, in the RF-C7 training the hip, knee, and ankle angles take on greater significance than their angular velocities. In both cases, the ankle angular velocity has lower importance than the hip and knee kinematics.

According to the results, vertical acceleration has a low impact on the training of the models compared to the lower limb kinematics. In the FNN-C7 model, the vertical acceleration has the second lowest importance, and in the RF-C7 model, it has the lowest relevance.

### 3.2 Validation and test results

**FIGURE 5:**
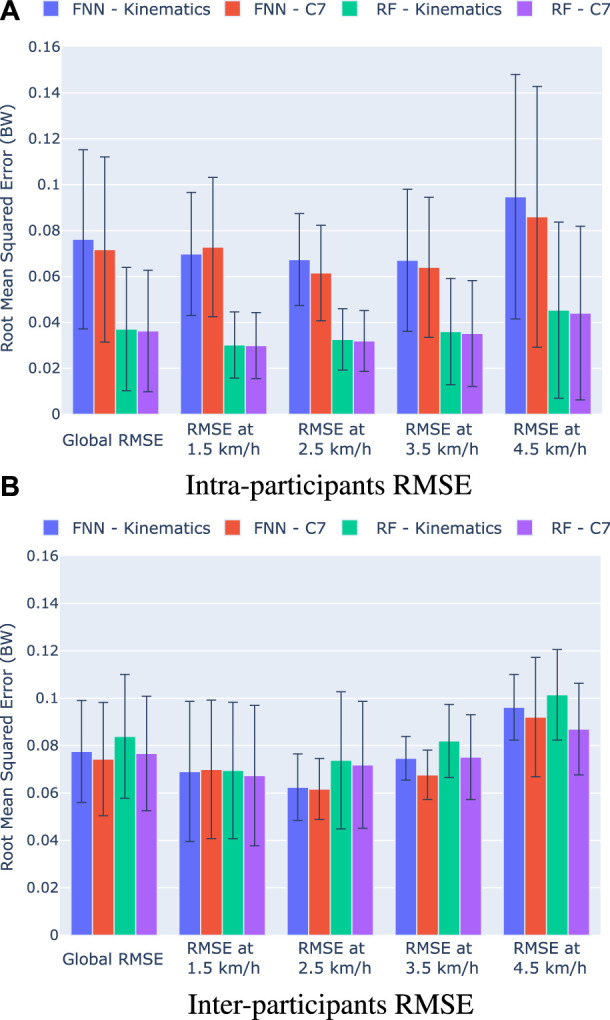
Mean RMSE (BW) and standard deviation for each training of the FNN and RF models. The graphs show the global RMSE and the RMSE per gait speed with **(A)** The intra-participant data **(B)** the inter-participant data.

Once we performed the two established pieces of training (Kinematics, C7), we evaluated the accuracy of the models with the validation and test data. We have calculated the RMSE for each of the registered steps to evaluate the performance of the models with the intra-participants and inter-participants. The mean and standard deviations of the RMSE errors are shown in Figure 5, where Figure 5A show the results with the intra-participants, and Figure 5B the results with the inter-participants. In addition, Table 1 and Table 2 show respectively the validation and test results.

**TABLE 1:** Validation mean (standard deviation) RMSE (BW) for each training of the FNN and RF models. The global RMSE errors for each model are shown. In addition, the table collects the RMSE errors obtained for each gait speed.

	FNN - Kinematics (a)	FNN - C7 (b)	RF - Kinematics (c)	RF - C7 (d)
Global	0.076 (0.039) ***^c,*d*^	0.072 (0.040) ***^c,*d*^	0.037 (0.027) ***^a,*b*,*d*^	0.036 (0.026) ***^a,*b*,*c*^
1.5 km/h	0.070 (0.027) ***^c,*d*^	0.073 (0.030) ***^c,*d*^	0.030 (0.014) ***^a,b^*^d,^	0.030 (0.015) ***^a,b^*^c,^
2.5 km/h	0.067 (0.020) ***^c,*d*^	0.062 (0.020) ***^c,*d*^	0.033 (0.013) ***^a,*b*,*d*^	0.032 (0.013) ***^a,*b*,*c*^
3.5 km/h	0.067 (0.030) ***^c,*d*^	0.064 (0.030) ***^c,*d*^	0.036 (0.023) ***^a,*b*,*d*^	0.035 (0.023) ***^a,*b*,*c*^
4.5 km/h	0.095 (0.050) ***^c,*d*^	0.086 (0.056) ***^c,*d*^	0.045 (0.038) ***^a,*b*,*d*^	0.044 (0.037) ***^a,*b*,*c*^

Statistical differences are represented by * (*p* < = 0.05), ** (*p* < = 0.01), *** (*p* < = 0.001), and **** (*p* < = 0.0001).

**TABLE 2:** Test mean (standard deviation) RMSE (BW) for each training of the FNN and RF models. The global RMSE errors for each model are shown. In addition, the table collects the RMSE errors obtained for each gait speed.

	FNN - Kinematics	FNN - C7	RF - Kinematics	RF - C7
Global	0.077 (0.022)	0.074 (0.028)	0.083 (0.026)	0.077 (0.024)
1.5 km/h	0.069 (0.030)	0.070 (0.029)	0.070 (0.029)	0.067 (0.030)
2.5 km/h	0.062 (0.014)	0.062 (0.013)	0.074 (0.029)	0.072 (0.027)
3.5 km/h	0.075 (0.010)	0.068 (0.010)	0.082 (0.012)	0.075 (0.018)
4.5 km/h	0.096 (0.014)	0.092 (0.025)	0.102 (0.019)	0.087 (0.019)

No statistical differences were found.

The statistical analysis showed significant differences between the performance of the models with the validation data for each gait speed (Friedman test *p* < 0.0001 for all speeds). In the pairwise comparison, the results show that there is not a significant difference in the accuracy of the models when the users’ vertical acceleration is included as an input of the model for each gait speed (*p* > 0.05). However, the inclusion of the vertical acceleration seems to reduce significantly the global error when using the FNN models (*p* < 0.05). When we compare the FNN and RF performance, we see a significant reduction of the RMSE when we employ the RF models with the validation data for the global RMSE errors and for all gait speeds. When we analyze the effect of the vertical acceleration, despite there is not an statistical significance, we observe a reduction of the RMSE errors in the RF training when the vertical acceleration is included, specially for the gait speeds of 3.5 and 4.5 km/h. This behavior can also be seen when using the FNN models. However, it should be noted that, when we employ a FNN model, the inclusion of the vertical acceleration is detrimental to the model performance at 1.5 km/h.

The analysis of the RMSE errors obtained with the inter-participants shows that there is not a significant difference between the accuracy of the models (Friedman test for global RMSE *p* = 0.24, at 1.5 km/h *p* = 0.80, at 2.5 km/h *p* = 0.80, at 3.5 km/h *p* = 0.17, and at 4.5 km/h *p* = 0.24). Nevertheless, it can be observed that there is also a reduction of errors in both models when the vertical acceleration is included. Moreover, we can observe similar results in terms of RMSE errors if we compare the FNN and RF models.

The results suggest that the FNN-C7 model and the RF-C7 model have higher accuracy in terms of RMSE errors. Hence, both models are analyzed deeply in the next section.

### 3.3 Validation and test vGRF signal reconstruction

**FIGURE 6:**
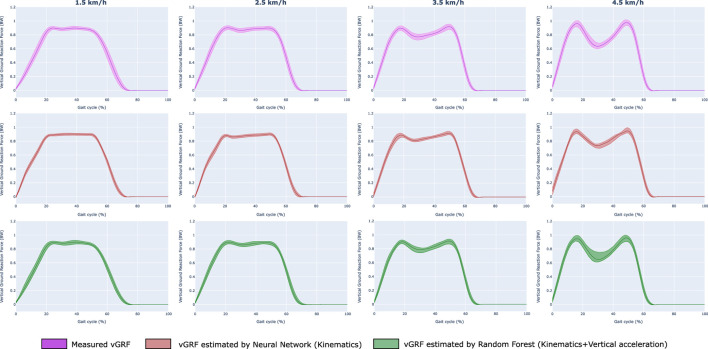
Epoch graphs with the measured and estimated vGRF (intra-participants and inter-participants) according to the cycle gait (%) for different gait speeds. The median data value has been represented, and the values between the first and third quartiles have been colored. The first row collects the measured vGRF, the second row the estimated vGRF with the selected FNN model (Training 2, C7), and the third row the estimated vGRF with the selected RF model (Training 2, C7). The vGRF representations include all participant users (intra-participants and inter-participants).

In Figure 6 we have represented the measured vGRF, the vGRF estimated with the FNN-C7 model, and the vGRF estimated with the RF-C7 model. In these graphs, the median value of the vGRF is represented during the gait cycle, and the shaded areas correspond to the vGRF values between the first and third quartiles.

**FIGURE 7:**
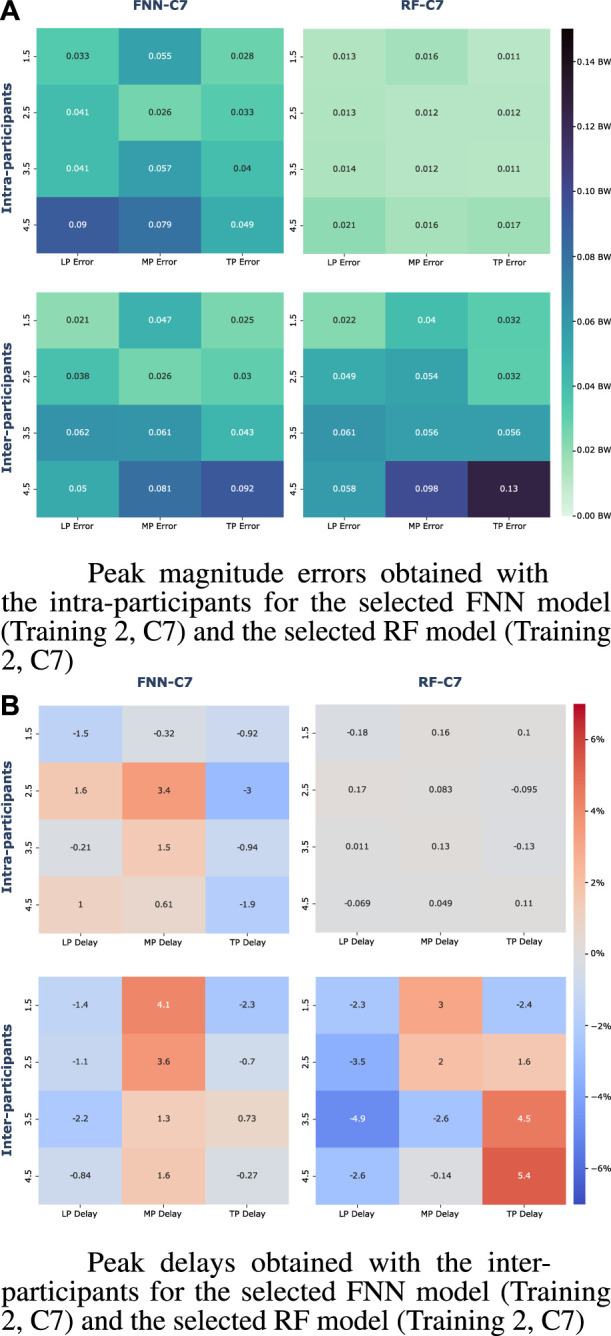
Heatmaps with the vGRF peak errors for gait speeds of 1.5 km/h, 2.5 km/h, 3.5 km/h, and 4.5 km/h. **(A)** Mean magnitude errors of the vGRF peaks (BW) **(B)** Mean delays of the vGRF peaks (%).

Table 3 and Table 4 collects the correlation (*ρ*) and NRMSE between the measured and estimated vGRF with the selected models for the intra-participants and the inter-participants, respectively. Figure 7 shows heatmaps with the mean of the peaks magnitude errors and delays with the intra-participants and inter-participants. Moreover, the Supplemental Data collects detailed information about the magnitude error and delay of the characteristic peaks, and the pairwise comparison results between the selected models (FNN-C7 and RF-C7).

**TABLE 3:** Correlation (*ρ*) and normalized RMSE (NRMSE) results obtained between the measured and estimated vGRF with the intra-participants using the FNN-C7 and RF-C7 models.

	Gait speed (km/h)	*ρ*	NRMSE
FNN-C7	1.5	0.9817	0.0483
	2.5	0.9886	0.0399
	3.5	0.9847	0.0449
	4.5	0.9676	0.0651
RF-C7	1.5	0.9988	0.0118
	2.5	0.9987	0.0127
	3.5	0.9980	0.0155
	4.5	0.9966	0.0210

**TABLE 4:** Correlation (*ρ*) and normalized RMSE (NRMSE) results obtained between the measured and estimated vGRF with the inter-participants using the FNN-C7 and RF-C7 models.

	Gait speed (km/h)	*ρ*	NRMSE
FNN-C7	1.5	0.9777	0.0718
	2.5	0.9876	0.0560
	3.5	0.9859	0.0604
	4.5	0.9705	0.0853
RF-C7	1.5	0.9776	0.0716
	2.5	0.9797	0.0708
	3.5	0.9797	0.0691
	4.5	0.9746	0.0792

With the intra-participants, the results show *ρ* values in the vGRF reconstruction between 0.9676 and 0.9886 with the FNN-C7 model, and coefficients between 0.9966 and 0.9988 with the RF-C7 model. In addition, the results show NRMSE values between 0.0399 BW and 0.0651 BW with the FNN-C7 model, and values between 0.0127 BW and 0.0210 BW with the RF-C7 model. When the vGRF is predicted with the inter-participants the correlation is reduced, with *ρ* values between 0.9705 and 0.9876 with the FFN-C7 model, and values between 0.9746 and 0.9849 with the RF-C7 model. According to the NRMSE results, values are between 0.0560 BW and 0.0853 BW with the FNN-C7 model, and values are between 0.0691 BW and 0.0792 BW with the RF-C7 model.

The analysis of the vGRF peaks shows statistical differences with the intra-participants for all the characteristic peaks (*p* < 0.0001). The results collected in Figure 7 show that all the peak magnitude errors obtained with the RF-C7 model are lower than errors obtained with the FNN-C7 model. The RF-C7 achieves magnitude errors lower than 0.0210 BW for the LP, 0.0161 BW for the MP, and 0.0171 BW for the TP. Otherwise, the FNN-C7 model errors are up to 0.0900 BW in the LP, 0.0793 BW for the MP, and 0.0491 BW for the TP. By contrast, when the models are evaluated with the inter-participant data, the statistical analysis has shown that there are differences in the prediction of the peak magnitude errors between models except for the MP at 3.5 km/h (*p* = 0.1825) and TP at 4.5 km/h (*p* = 0.0834). In the case of the FNN-C7 model, we can find errors between to 0.0206 BW and 0.0619 BW in the LP, between 0.0263 BW and 0.0810 BW in the MP, and between 0.0249 BW and 0.0922 BW in the TP. By contrast, the obtained errors with the RF-C7 model are between 0.0225 BW and 0.0611 BW in the LP, between 0.0399 BW and 0.0983 BW in the MP, and between 0.0316 BW and 0.1256 BW in the TP.

The analysis of the delay of the predicted vGRF peaks with the intra-participants shows that the lags achieved by the RF-C7 model are closely 0%, with mean lags lower than 0.18% (advance in LP at 1.5 km/h), while the timing errors with the FNN-C7 range from −0.21% to 1.6% in the LP, from −0.3% to 3.4% in the MP, and −0.92% to −3% in the TP. Furthermore, an increase in the timing errors can be appreciated for both models when they are evaluated with the inter-participant data. The timing errors obtained with the FNN-C7 model range from −0.84% to −2.2% in the LP, from 1.3% to 4.1% in the MP, and −2.3%–0.73% in the TP, while the RF-C7 model errors range from −2.3% to −4.9% in the LP, from −2.6% to 3% in the MP, and −2.4% to 5.4 in the TP estimation.

## 4 Discussion

This work presents a method to estimate vGRF using diverse inertial sensors placed over the lower limbs and the C7 vertebra.

The IMUs placed on the hip, thigh, shank, and foot are used to measure the flexion/extension angles of the hip, knee, and ankle joints. For this purpose, the method developed by T. Seel et al. was used to estimate the joint angles by means of the accelerometer and gyroscope data provided by the sensors (Seel et al., 2014). One of the main advantages of this method is that the position and orientation of the IMUs do not influence the estimation of joint angles. Therefore, we can assume that variations in sensor placement will not affect the error made in the estimation of vGRF. Furthermore, the calibration method of this algorithm is simple and does not require precise movements, as the calibration is based on walking and performing random movements with the legs for a short period.

We have trained and evaluated the performance of two machine learning models in order to estimate the vGRF during gait. First, we have calculated the feature importance for the FNN-C7 and RF-C7 models to understand the relevance of each input (Figure 4). For both models, the results show that the gait cycle has the biggest influence on the training of the model, which suggests that the proposed method for estimating vGRF has a strong dependence on time. This makes sense since the values of angles, angular velocities, and vertical acceleration can take the same value for different instants of the gait cycle, but the vGRF takes different values. Moreover, from the results, it also can be extracted that hip and knee kinematics have a bigger importance than ankle kinematics.

We introduced that vertical acceleration is strongly related to the vGRF. However, when the feature importance is calculated, we can observe that vertical acceleration has a small influence on the model training compared to the kinematics of the lower limbs. Despite the vertical acceleration seems to have low relevance in the models, the results suggest that this input could modify the performance of the FNN and RF models (Figure 5; Table 1; Table 2). Although no statistical differences were found, according to the validation and test results represented in Figure 5, we can observe that the vertical acceleration appears to improve the performance in the FNN and RF models in terms of RMSE. By contrast, the RMSE obtained with the FNN models when the vertical acceleration is included seems to increase. Hence, it could be concluded that vertical acceleration is not crucial to estimate the vGRF with the method proposed if we are able to measure the lower limb kinematics, but it might help to improve the performance of certain models.

In addition, when we compare the performance of the four models trained (FNN-Kinematics, FNN-C7, RF-Kinematics, and RF-C7) some differences can be observed in terms of RMSE. When we employ the validation data (i.e., data from the intra-participants), the RMSE obtained with the RF models (0.030–0.045 BW) is almost half the RMSE of obtained with the FNN models (0.067–0.095 BW). However, when we evaluate the models with the inter-participants, these differences are not statistically significant, and we obtain similar RMSE with the four models. It is usual to obtain an increase in the error with inter-participant data, but the behavior of the RF models might indicate that they tend to overfit more than the FNN models, which is a known problem on algorithms based on decision trees (Dietterich, 1995). Despite the overfitting, the results suggest that the RF models achieve similar accuracy, with a global RMSE of 0.077 BW for the RF-C7 model, and 0.074 BW for the FNN-C7 model, which are the best FNN and RF models in terms of RMSE.

If we analyze further the FNN-C7 and the RF-C7, Table 4 shows that the measured and estimated vGRF are strongly correlated for both intra-participants and inter-participants. In the same manner, as can be seen in the results obtained for RMSE, RF-C7 achieves higher correlation coefficients than the one achieved by the FNN-C7 model with the intra-participant data, but with the inter-participants, the FNN-C7 correlations are higher than the achieved by the RF-C7 for gait speeds between 1.5 and 4.5 km/h. The NRMSE results show the same behavior, where the FNN-C7 achieves higher errors than the RF-C7 model. However, with the inter-participant data, the NRMSE for 2.5 and 3.5 km/h are lower with the FNN-C7, while the RF-C7 obtains lower errors for 1.5 and 4.5 km/h.

The estimated vGRF signals shown in Figure 6 indicate that both selected models can replicate the measured vGRF. It can be observed that the FNN-C7 and the RF-C7 models can differentiate the cycle gait phases, as the graphs show that they might estimate the characteristic peaks and the swing phase (vGRF = 0 BW).

It must be noted that the magnitude and timing of the vGRF peaks are important parameters to perform gait analysis. For this reason, these peaks are deeply analyzed (Figure 7, Supplemental Data). The RF-C7 model has shown a significantly higher accuracy than the FNN-C7 model according to the magnitude errors in intra-participant users for all the peaks at each gait speed. Nevertheless, the errors of the peaks obtained with the inter-participants are similar between both models. We must remark that the errors obtained with the FNN-C7 in the LP at 1.5, 2.5, and 4.5 km/h, and the TP at 1.5 and 2.5 km/h are lower with the inter-participant data than the intra-participants. Although, it can be observed that the errors obtained with RF-C7 for the inter-participants are higher than the intra-participants for all the peaks and gait speeds. These results would be in agreement with what was seen previously with the RMSE and could be due to the overfitting of the RF models.

Concerning the timing errors of the vGRF peaks, the RF-C7 model presents a high accuracy in the timing prediction of the peaks at all gait speeds with the intra-participant data, with a maximum lag of 0.18% of the cycle gait. On the other hand, the FNN-C7 model appears to make a larger error at the peak timing, especially in the MP with mean delays up to 3.4%. The results obtained for the inter-participants show larger delays and advances in the estimation of the peaks with respect to the measured vGRF, specially with the RF-C7 model. We must mention that all the mean lags obtained with the RF-C7 are greater than the FNN-C7 except for the MP peak at 1.5 and 2.5 km/h.

It must be highlighted that we can assume that both FNN-C7 and RF-C7 models are robust and they have good accuracy to estimate the vGRF by using the kinematics of the lower limbs and the vertical acceleration. This conclusion can be extracted since the RMSE and *ρ* of our models are in a similar range to studies made by other authors when the vGRF was estimated during gait. In (Oh et al., 2013) the authors obtained an RMSE of around 0.066 BW and *ρ* of 0.991 with intra-participants; in (Choi et al., 2013) the authors obtained an RMSE of around 0.074 BW and *ρ* of 0.99 with intra-participants; in (Jiang et al., 2020) the authors obtained an RMSE of 0.02 BW and *ρ* of 1.00 with intra-participants, and an RMSE of 0.10 BW and *ρ* of 0.97 with inter-participants. Despite the RF model trained by Jiang et al. seems to achieve a higher accuracy than our models with intra-participants, the results with inter-participants suggest that our RF and FNN models achieve a lower RMSE and higher *ρ*, so they would have a higher generalization than those proposed by Jiang et al.

It should be noted that, although the FNN-C7 model and the RF-C7 model achieve good accuracy compared to previous works, the results show differences between them. First, the mean of the RMSE obtained with the intra-participants is lower with the RF-C7 model, but the global RMSE achieved by the FNN-C7 is lower than the RF models. However, the calculated *ρ* and NRMSE show that coefficients and errors obtained are lower at 1.5 and 4.5 km/h with the RF-C7, but lower at walking speeds of 2.5 and 3.5 km/h with the FNN-C7. Finally, the mean magnitude errors of the characteristic peaks show similar results for the inter-participants. Furthermore, the FNN-C7 seems to achieve higher performance than the RF-C7 in estimating the timing of the peaks. Therefore, based on these differences, we could assume that the FNN-C7 model overcomes the accuracy of the trained RF-C7 model.

## 5 Conclusion

This work aims to present a method to estimate the vGRF using wearable IMUs. We used inertial sensors to estimate the kinematics of the lower limbs and the vertical acceleration of the users, which were employed to train two types of machine learning models: Feedforward Neural Networks (FNN) and Random Forest (RF).

The results show that the trained models have a big influence on the temporal variable (cycle gait), followed by the hip, knee, and ankle kinematics. The inclusion of vertical acceleration has a small influence on the training of the FNN and RF models compared to the rest of the inputs. However, the results suggest that the inclusion of vertical acceleration can modify the performance of the model. In the case of the FNN and RF models, the inclusion of this feature appears to increase the performance.

Moreover, we can assume that the method proposed to estimate the vGRF has good accuracy for biomechanical analysis, as the estimate of vGRF, including its peaks, is in a similar range of accuracy to that reported in other studies, and even the results suggest that a smaller error is obtained in the estimation of VGRF. Furthermore, the RMSE, *ρ*, and the errors in the characteristic peaks suggest that the FNN-C7 model achieves the highest accuracy of the trained machine learning models.

## Data Availability

The raw data supporting the conclusion of this article will be made available by the authors, without undue reservation.
