# Supplementary material for: Estimating vertical ground reaction forces during gait from lower limb kinematics and vertical acceleration using wearable inertial sensors
Source: Front Bioeng Biotechnol. 2023 Sep 29;11:1199459. doi: 10.3389/fbioe.2023.1199459 (PMC10570513; doi:10.3389/fbioe.2023.1199459)
Supplement: Supplementary file 1 [file DataSheet1.PDF]

Supplementary Material

1 SUPPLEMENTARY TABLES

These supplementary Tables present detailed information about the analysis of the estimated characteristic peaks of the vGRF. Table S1 and Table S2 collect the mean magnitude error and delay of the LP, MP, and TP peaks, using the FNN-Kinematics and RF-C7 models with intra-participant and inter-participant data, respectively. Furthermore, the pairwise comparison values between the FNN and the RF errors are shown.

Table S1. Results obtained in the vGRF estimation with the selected FNN and RF models employing data from the intra-participants. The table collects the mean peak magnitude errors, and the mean peak delays between the measured and estimated vGRF for each gait speed. Moreover, the p-values (Wilcoxon-Pratt test) for the pairwise comparison between the selected models are shown.

	**Gait Speed (km/h)**	**FNN-C7**	**RF-C7**	**p-value**
**LP Error (BW)**	1.5	0.0334 ± 0.0256	0.0128 ± 0.0075	p<0.0001
	2.5	0.0407 ± 0.0283	0.0132 ± 0.0080	p<0.0001
	3.5	0.0409 ± 0.0467	0.0139 ± 0.0138	p<0.0001
	4.5	0.0900 ± 0.1026	0.0210 ± 0.0286	p<0.0001
**MP Error (BW)**	1.5	0.0562 ± 0.0561	0.0161 ± 0.0147	p<0.0001
	2.5	0.0262 ± 0.0330	0.0119 ± 0.0097	p<0.0001
	3.5	0.0566 ± 0.0376	0.0120 ± 0.0102	p<0.0001
	4.5	0.0793 ± 0.0596	0.0158 ± 0.0184	p<0.0001
**TP Error (BW)**	1.5	0.0279 ± 0.0195	0.0110 ± 0.0062	p<0.0001
	2.5	0.0331 ± 0.0205	0.0123 ± 0.0072	p<0.0001
	3.5	0.0396 ± 0.0304	0.0114 ± 0.0117	p<0.0001
	4.5	0.0491 ± 0.0428	0.0171 ± 0.0209	p<0.0001
**LP Delay (%)**	1.5	-1.51 ± 6.47	-0.19 ± 2.53	p<0.0001
	2.5	1.57 ± 5.97	0.17 ± 2.96	p<0.0001
	3.5	-0.20 ± 4.04	0.01 ± 2.34	0.0772
	4.5	0.99 ± 2.05	-0.07 ± 1.36	p<0.0001
**MP Delay (%)**	1.5	-0.32 ± 6.80	0.16 ± 3.12	0.4353
	2.5	3.43 ± 5.10	0.08 ± 3.00	p<0.0001
	3.5	1.47 ± 4.98	0.13 ± 2.53	p<0.0001
	4.5	0.61 ± 3.38	0.05 ± 1.94	p<0.0001
**TP Delay (%)**	1.5	-0.92 ± 6.24	0.10 ± 2.31	p<0.0001
	2.5	-3.00 ± 7.23	-0.09 ± 3.25	p<0.0001
	3.5	-0.93 ± 2.75	-0.13 ± 1.73	p<0.0001
	4.5	-1.89 ± 2.43	0.11 ± 1.42	p<0.0001

Table S2. Results obtained in the vGRF estimation with the selected FNN and RF models employing data from the inter-participants. The table collects the mean peak magnitude errors, and the mean peak delays between the measured and estimated vGRF for each gait speed. Moreover, the p-values (Wilcoxon-Pratt test) for the pairwise comparison between the selected models are shown.

	Gait Speed (km/h)	FNN-C7	RF-C7	p value
**LP Error (BW)**	1.5	0.0206 ± 0.0161	0.0225 ± 0.0162	0.0142
	2.5	0.0382 ± 0.0254	0.0492 ± 0.0305	p<0.0001
	3.5	0.0619 ± 0.0362	0.0611 ± 0.0315	p<0.0001
	4.5	0.0499 ± 0.0408	0.0579 ± 0.0375	p<0.0001
**MP Error (BW)**	1.5	0.0467 ± 0.0583	0.0399 ± 0.0540	p<0.0001
	2.5	0.0263 ± 0.0194	0.0535 ± 0.0296	p<0.0001
	3.5	0.0690 ± 0.0306	0.0563 ± 0.0315	0.1825
	4.5	0.0810 ± 0.0410	0.0983 ± 0.0418	p<0.0001
**TP Error (BW)**	1.5	0.0249 ± 0.0377	0.0319 ± 0.0393	p<0.0001
	2.5	0.0302 ± 0.0177	0.0316 ± 0.0221	p<0.0001
	3.5	0.0435 ± 0.0265	0.0564 ± 0.0462	p<0.0001
	4.5	0.0922 ± 0.0386	0.1256 ± 0.0466	0.0834
**LP Delay (%)**	1.5	-1.37 ± 6.72	-2.34 ± 5.21	0.0001
	2.5	-1.12 ± 7.17	-3.52 ± 6.35	p<0.0001
	3.5	-2.21 ± 5.30	-4.88 ± 5.49	p<0.0001
	4.5	-0.84 ± 3.34	-2.63 ± 3.15	p<0.0001
**MP Delay (%)**	1.5	4.14 ± 6.95	3.01 ± 6.79	0.0008
	2.5	3.56 ± 5.48	2.00 ± 6.25	p<0.0001
	3.5	1.267 ± 4.04	-2.57 ± 4.61	p<0.0001
	4.5	1.56 ± 2.37	-0.14 ± 3.66	p<0.0001
**TP Delay (%)**	1.5	-2.33 ± 7.40	-2.39 ± 6.40	0.7777
	2.5	-0.70 ± 6.61	1.60 ± 7.71	p<0.0001
	3.5	0.73 ± 3.27	4.51 ± 4.69	p<0.0001
	4.5	-0.27 ± 2.41	5.35 ± 2.88	p<0.0001
